# Analysis of Faecal Microbiota and Small ncRNAs in Autism: Detection of miRNAs and piRNAs with Possible Implications in Host–Gut Microbiota Cross-Talk

**DOI:** 10.3390/nu14071340

**Published:** 2022-03-23

**Authors:** Federica Chiappori, Francesca Anna Cupaioli, Arianna Consiglio, Noemi Di Nanni, Ettore Mosca, Vito Flavio Licciulli, Alessandra Mezzelani

**Affiliations:** 1 Institute for Biomedical Technologies, National Research Council of Italy, 20054 Segrate, Italy; federica.chiappori@itb.cnr.it (F.C.); francesca.cupaioli@itb.cnr.it (F.A.C.); noemi.dinanni@itb.cnr.it (N.D.N.); ettore.mosca@itb.cnr.it (E.M.); 2 Institute for Biomedical Technologies, National Research Council of Italy, 70126 Bari, Italy; arianna.consiglio@ba.itb.cnr.it (A.C.); flavio.licciulli@ba.itb.cnr.it (V.F.L.)

**Keywords:** autism spectrum disorders, host–gut microbiota cross-talk, gene-environment interaction, multi-omics, microbiome, mycobiome, piRNAs, microRNAs

## Abstract

Intestinal microorganisms impact health by maintaining gut homeostasis and shaping the host immunity, while gut dysbiosis associates with many conditions, including autism, a complex neurodevelopmental disorder with multifactorial aetiology. In autism, gut dysbiosis correlates with symptom severity and is characterised by a reduced bacterial variability and a diminished beneficial commensal relationship. Microbiota can influence the expression of host microRNAs that, in turn, regulate the growth of intestinal bacteria by means of bidirectional host-gut microbiota cross-talk. We investigated possible interactions among intestinal microbes and between them and host transcriptional modulators in autism. To this purpose, we analysed, by “omics” technologies, faecal microbiome, mycobiome, and small non-coding-RNAs (particularly miRNAs and piRNAs) of children with autism and neurotypical development. Patients displayed gut dysbiosis related to a reduction of healthy gut micro- and mycobiota as well as up-regulated transcriptional modulators. The targets of dysregulated non-coding-RNAs are involved in intestinal permeability, inflammation, and autism. Furthermore, microbial families, underrepresented in patients, participate in the production of human essential metabolites negatively influencing the health condition. Here, we propose a novel approach to analyse faeces as a whole, and for the first time, we detected miRNAs and piRNAs in faecal samples of patients with autism.

## 1. Introduction

Autism spectrum disorders (ASD) refers to a group of complex neurodevelopmental conditions whose core symptoms are a deficit in communication and social interaction, restricted interests, and repetitive behaviours (https://www.who.int/news-room/fact-sheets/detail/autism-spectrum-disorders, accessed on 26 November 2021). Comorbidities, such as mental retardation, epilepsy, anxiety, sensory, sleep, and gastrointestinal disorders, as well as food selectivity often occur in ASD [1,2,3]. ASD manifests during the first years of age, and in the last decades, its prevalence has continued to increase, reaching the frequency of one in 54 children aged eight years in the USA in the 2016 [4]. ASD aetiology is multifactorial and has not yet completely elucidated although the interaction between genetic susceptibility and environmental factors is emerging as the most consistent cause of ASD development and severity [2,5,6]. To date, hundreds of risk genes have been associated with ASD as reported in Simons Foundation Autism Research Initiative (SFARI) database (https://gene.sfari.org, accessed on 2 December 2021), and gut dysbiosis is reputed as the most impactful among environmental factors [3].

Gut microbiota assembles more than 100 trillion microorganisms that harbour over 3 million genes (the human genome consists of about 23,000 genes), producing thousands of molecules and metabolites [7]. It includes bacteria and archaea that represents over 99% of genes and fungi and viruses with only 0.1% of genes [8].

The most studied microorganisms are prokaryotes, while the remaining domains are still poorly investigated. Microorganisms contribute to the digestion, synthesis, and absorption of many nutrients and metabolites, and they compete with pathogens, maintain the intestinal barrier integrity, and impact cell signalling pathways [7,9]. Moreover, a bidirectional communication system, the so-called “microbiota–gut–brain axis”, which connects the central and the enteric nervous system by biochemical signalling, is able to regulate cognitive skills and behaviour [10,11]. Indeed, the microbial community has a key role in orchestrating the cross-talk via epigenetics, metabolites, hormones, and afferent nerves [9,12,13]. This interaction exerts a profound influence on key neurodevelopmental processes, including neurogenesis, myelination, glial cell function, synaptic pruning, and blood–brain barrier function and permeability. The gut–brain axis also modulates neurotransmission and neuroinflammation in adults [12], and its imbalance is involved in neuropsychiatric disorders, including ASD [14,15].

Gut dysbiosis, the imbalance in the taxonomic composition of microbiota in general, increases intestinal permeability and inflammation, leading to abnormal molecule trafficking and potential translocation of intestinal microorganisms to the bloodstream [16]. Gut dysbiosis associates with many diseases and neurologic conditions, including ASD, and strongly correlates with the severity of its symptoms [17,18]. Bacterial composition has been deeply investigated in ASD, while there is a lack of mycobiome studies, leading to a need for a consistent and complete microbial profile associated to ASD [19]. Overall, these studies return a lower microbial diversity that, individually, represents a form of dysbiosis since they have been associated with several conditions and aging [10,18]. In humans, the commensal bacterial community of healthy gut includes *Firmicutes* (mainly including *Clostridium, Enterococcus, Lactobacillus,* and *Faecalibacterium* genera), *Bacteroidetes* (including *Bacteroides* and *Prevotella* genera), *Actinobacteria, Proteobacteria, Fusobacteria*, and *Verrucomicrobia* phyla, with *Firmicutes* and *Bacteroidetes* representing 90% of gut microorganism [20]. The dominant families are *Prevotella, Bacteroidaceae*, and *Ruminococcaceae* [21]. Lower levels of *Bifidobacterium* and higher levels of *Lactobacillus, Clostridium, Bacteroidetes, Desulfovibrio, Caloramator,* and *Sarcina* were reported in ASD compared to healthy controls [22]. Chen and colleagues confirmed a *Clostridium* and *Bifidobacterium* trend and detected a significant decrease of *Prevotella, Blautia,* and *Dialister* [18].

Gut mycobiota, the community of commensal eukaryotes, have received much less attention than bacteria to date. Furthermore, its profiling is complicated due to a complex taxonomic annotation of fungi [23,24]. The mycobiota interacts with commensal bacteria and the host and maintains the homeostasis of microbiota, thus influencing gut health [25]. Moreover, the mycobiota influence the host immunological responses, modulating the local inflammatory system [26,27]. Infants’ gut mycobiota is dominated by *Malasseziales* until six months of age, most likely acquired through lactation. Then, during weaning, it undergoes a strong change although it maintains a low microbial complexity and diversity and becomes dominated by *Candida* (particularly *C. albicans*), *Saccharomyces* (particularly *S. cerevisiae*), *Penicillium, Aspergillus, Cryptococcus, Malassezia* (particularly *M. restricta*), *Cladosporium, Galactomyces, Debaryomyces*, and *Trichosporon* [28,29,30]. The few studies regarding mycobiota in ASD reported an increase of *Candida genus*, mainly *C. Albicans,* and a decrease of *Aspergillus* and *Penicillium* [31,32,33]. *C. Albicans* represents about 80% of yeast in ASD with a respective 20% of neurotypical controls [32]. It has been demonstrated that the host shapes the gut microbial community, releasing microRNAs (miRNAs) in the intestinal lumen [34]. Indeed, the intestinal epithelial cells and Hopx-positive cells (quiescent stem cells) can modulate their own gut microbiota by releasing extracellular vesicles (EVs) containing miRNAs into the gastrointestinal tract. Interestingly, these miRNAs can enter bacteria and act at the DNA or RNA level to affect gene expression and control the microbial growth [34]. By means of this mechanism, the host can regulate the composition of its own microbiota that, in return, can influence host miRNA expression via MyD88-dependent pathway [11,35,36]. Furthermore, microbiota-derived metabolites and microbiota-derived EVs also participate in the host–microbiota cross-talk regulating gene expression and intestinal homeostasis of the hosts [11,37,38]. The potential cross-talk between faecal microbiota and miRNA expression in pathological conditions has been identified in inflammatory bowel disease and colorectal cancer, also underlying their potential clinical relevance as biomarkers and therapeutic targets [35,39]. For instance, miR-515-5p and miR-1226-5p induce the growth of *Fusobacterium nucleatum* and *E. coli*, respectively [40], while commensal microbiota-induced miR-21-5p over-expression is involved in intestinal permeability via ARF4 [40], thus representing a therapeutic target to restore an intestinal barrier.

Recently, a study found an association between salivary miRNAs and salivary microbiota dysregulation in ASD [41], but none of the studies reported host miRNA-gut microbiota interaction in this condition. PiRNAs are small ncRNA that epigenetically and post-transcriptionally silence the expression of transposable elements integrated in eukaryotic genomes [42]. At present, no piRNAs in stool samples are available either in healthy or in pathological conditions.

In this scenario, it emerges that microbes and small non-coding RNA (sncRNA) in faecal samples should be considered and studied as a whole to comprehend how microbial strains interact among each other and with the host. In this pilot study, we defined, through “omics” technologies, the faecal micro- and mycobioma profile as well as the sncRNA profile of a small group of individuals with ASD and neurotypical controls. Our aim was to find markers of ASD among stool microbial and transcriptional modulators for the possible relationship between them and the host. We applied a bioinformatics approach to correlate gut bacteria and fungi composition with host miRNAs and piRNA expression for the first time in stool samples from patients with ASD and attempted to highlight possible mechanisms of microbiota-host bidirectional cross-talk in ASD. 

## 2. Materials and Methods

### 2.1. Subjects

A total of 12 individuals, 6 with ASD (5 males and 1 female; age 6–17) and 6 neurotypical controls (Ctrl) (3 males and 3 females; age 10–20), were recruited for this study. The diagnoses for ASD are: three, autism; one, high-functioning autism; one, autism with echolalia and motor stereotypies; and one, Asperger syndrome with stereotypies. Diagnosis were performed or revised according to DSM-5 [43]. 

### 2.2. Ethical Committees

The study was conducted in accordance with the Declaration of Helsinki, and approved by the Ethics Committee of Istituto San Vincenzo (protocol code 312, 28 December 2018). The Ethics Committee prepared the informed consent, including the instructions for sample collection; and enrolled the children. The collected informed consents were signed by the parents and/or legal guardians since the individuals were underage. All methods were performed in accordance with relevant guidelines and regulations regarding observational studies.

### 2.3. Sample Collection

Naturally evacuated stool samples were obtained from all individuals and collected by previously instructed parents. Stools were collected in stool nucleic acid collection and transport tubes, then returned refrigerated to the Institute for Biomedical Technologies, CNR. Samples were stored at –80 °C until RNA/DNA extraction.

### 2.4. DNA and SmallRNA Extraction from Stool

Total DNA was extracted from frozen stool samples (200 mg) using commercial kit and the relative protocol for pathogen detection (QiAamp DNA stool mini kit, Qiagen GmbH, Hilden, Germany) with minor modification, and then, pre-lysis mechanical grinding was performed to increase sample homogenization, microbial lysis, and DNA extraction strength. Zirconia beads (100 μm in diameter) were added to ASL buffer (300 mg/mL buffer) before incubation at 95 °C, and three cycles per 1 min in a bead beater were performed before thermal lysis. The DNA quality (280/260 ratio) was checked by NanoDrop 2000 spectrophotometer (Thermo Fisher Scientific, Wilmington, DE, USA) and quantity measured by Qubit dsDNA HS Assay Kit (Thermo Fisher Scientific, Wilmington, DE, USA). 

RNA was isolated from frozen stool samples (200 mg) using the commercial kit RNeasy Power Microbiome (Qiagen GmbH, Hilden, Germany) according to the manufacturer’s protocol. RNA quality was assessed by Agilent RNA 6000 Nano on Agilent 2100 Bioanalyzer system (Agilent Technologies, Santa Clara, CA, USA), and RNA concentration measured by Qubit RNA assay (Thermo Fisher Scientific, Wilmington, DE, USA).

### 2.5. 16S and 18S Sequencing

The 16S and 18S rDNA, V3-V4 and NS1-NS2 regions, were amplified (primers sequence for 16S, forward 5′-CCTACGGGNGGCWGCAG-3′ and reverse 5′-GACTACHVGGGTATCTAATCC-3′; primers sequence for 18S, forward 5′-GTAGTCATATGCTTGTCTC-3′ and reverse 5′-GGCTGCTGGCACCAGACTTGC-3′) from faecal DNA, and paired-end sequencing was performed on the Illumina MiSeq Flow cell V3, 2 × 300 bp, returning an average of 0.8 million reads per sample for 16S and 28,000 reads per sample for 18S.

### 2.6. SmallRNA Sequencing

Small RNA-sequencing libraries were generated directly from total RNA isolated from the stool samples and performed with the TruSeq Small RNA Library Preparation Kits (Illumina, Inc., San Diego, CA, USA) based on the manufacturer’s protocol. Libraries were sequenced on NextSeq 500 (Illumina, Inc., San Diego, CA, USA), 1 × 75 bp and 30 million reads per sample.

### 2.7. Metataxonomic Bioinformatics Analysis 

Metataxonomic analysis was performed in R (4.0.3) using Dada2 [44] pipeline using phyloseq package [45] against DADA2-formatted reference databases latest available version (July 2021), that is, Silva v138 [46] for 16S and Silva v132 [47] for 18S. DADA2 plug-in was used to filter, trim, dereplicate, merge, remove chimaeras, and assign taxonomy to all produced sequences to obtain the Operational Taxonomic Units (OTUs). Variance Stabilising Transformation was applied to normalise across samples on OTUs with DESeq2 package [48] as described by McMurdie and Holmes [45]. For each sample, the number of observed OTUs and the percentages of relative abundances of phyla, orders, classes, and families were determined. To evaluate statistically significant differences between ASD and controls (Ctrl) at genus level, the univariate DESeq2 method was used [45,48]. Default Wald test was applied in DESeq2, and significance threshold was set to *p*-value < 0.05 for the 16S analysis, while for the 18S analysis, all results were considered.

### 2.8. Small, Non-Coding RNA Data Analysis

SmallRNA reads were processed according to a custom bioinformatics pipeline that we developed [49]. Summarising the main pipeline steps, smallRNA reads were checked for quality control using FastQC package (http://www.bioinformatics.babraham.ac.uk/projects/fastqc, accessed on 22 July 2020), filtered, and then mapped against Arena-Idb [50], a reference database representing a comprehensive and non-redundant dataset of public ncRNA sequences and annotations, using Bowtie aligner [51], with one mismatch in the leftmost 20 bp of the read. In order to obtain reliable read counts and to fix the problem of multireads [52], we used the RSEM tool [53] for accurate expression estimations of identified ncRNAs.

An evident heterogeneity in the expressions of several individual references required an accurate management of the expression normalization step. A reference-free clustering of the sequences was performed with the SEED [54], an algorithm for clustering very large NGS sets. Sequences were joined into clusters that differ by up to three mismatches and three overhanging residues from their virtual centre. The cardinalities of the clusters resulted to be more stable (by showing higher correlations among the samples) and were used to compute the scaling factors of TMM normalization. Such factors were applied to normalise the ncRNA reference expression counts. Expression data were analysed with edgeR (https://bioconductor.org/packages/release/bioc/html/edgeR.html, accessed on 22 July 2020). EdgeR package applied Robinson and Smyth exact statistical methods for multigroup experiments [55,56]. The Benjamini–Hochberg multiplicity correction method was used on the *p*-values to control the false-discovery rate (FDR).

### 2.9. Identification of sncRNA Targets and Relative Pathways

All identified miRNA and piRNA were annotated, based on miRPathDB (https://mpd.bioinf.uni-sb.de/, accessed on 8 November 2021) [57] and piRNAdb (https://www.pirnadb.org/, accessed on 8 November 2021). In particular, only experimentally validated miRNA gene targets and predicted piRNA targets with the highest amount of overlapping alignments were considered for further analysis. Common gene targets between different miRNAs and/or piRNAs belonging to different samples were studied. Gene targets were annotated using KEGG and MSigDB-Hallmark gene sets (https://www.gsea-msigdb.org/gsea/msigdb/, accessed on 16 November 2021) using KEGGREST (https://bioconductor.org/packages/release/bioc/html/KEGGREST.html, accessed on 16 November 2021) and R function msigdbr (v7.4.1) (https://igordot.github.io/msigdbr/, accessed on 16 November 2021), respectively.

## 3. Results

### 3.1. Microbiota Analysis

Intestinal microbiota composition was evaluated analysing V3–V4 regions of 16S and NS1–NS2 regions of 18S from six ASD and six Ctrl stool samples. Overall, 9.5 million and 330,000 reads were obtained, with an average of 800,000 (±450,000) and 28,000 (±21,000) reads per sample for 16S and 18S, respectively. Reads converged into about 3000 (±850) and 60 (±20) OTU per sample on average for 16S and 18S, respectively.

**Figure 1:**
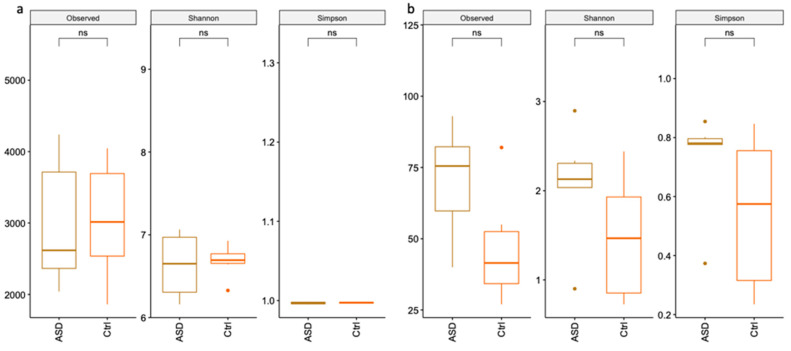
Alpha diversity analysis. Alpha diversity analysis with Shannon and Simpson indices of 16S (**a**) and 18S (**b**).

Analysis of 16S was performed considering all ASD samples against all Ctrl samples. As displayed in Figure 1a, no relevant differences can be evidenced between the two groups from the alpha diversity analysis. Instead, alpha diversity analysis, in particular Shannon and Simpson indices, of 18S samples (Figure 1b) highlight a major species diversity for ASD samples compared to controls.

#### 3.1.1. Bacteria Profiling: Metataxonomic Analysis

We identified *Actinobacteria*, *Bacteroidetes*, *Desulfobacterota*, *Firmicutes*, *Proteobacteria,* and *Verrucomicrobia* phyla. In accordance with literature, we found that *Bacteroides* and *Firmicutes* are the most represented phyla in the ASD and Ctrl faeces [7]. In detail, *Bacteroides* represent 42% in ASD and 14% in Ctrl, while *Firmicutes* are 44% in ASD and 85% in neurotypical. As previously reported, the *Bacteroidetes*/*Firmicutes* ratio was higher in ASD samples (0.79) than in Ctrl (0.38) [58,59,60].

**Figure 2:**
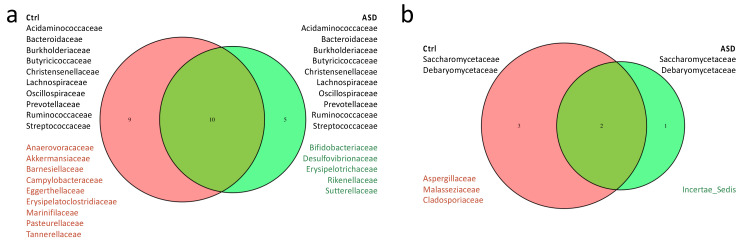
Venn diagrams of (**a**) microbiota and (**b**) mycobiota taxonomic analysis. In black, common families between ASD and Ctrl samples; in red, families mainly present in Ctrls; in green, families characterising the ASD group.

Metataxonomic comparative analysis was performed at family/genus level. ASD samples displayed a reduced microbiota variability compared to controls. In particular, 24 families, corresponding to 38 bacteria genera, were significantly detected in all samples; among these, nine families are prevalent in healthy samples and five in ASD (Figure 2). Considering differences in microbiota composition at genus level, ASD samples displayed a prevalence of *Bifidobacterium*, *Desulfovibrio*, *Coprococcus, Alistipes,* and *Sutterella*. Instead, in neurotypical samples, the most commonly represented genera are *Phascolarctobacterium*, *Akkermansia*, *Barnesiella*, *Enterorhabdus*, *Lachnospiraceae_NK4A136*, *Ruminococcus*, *Prevotellaceae_UCG-001,* and *Streptococcus*. 

**Figure 3:**
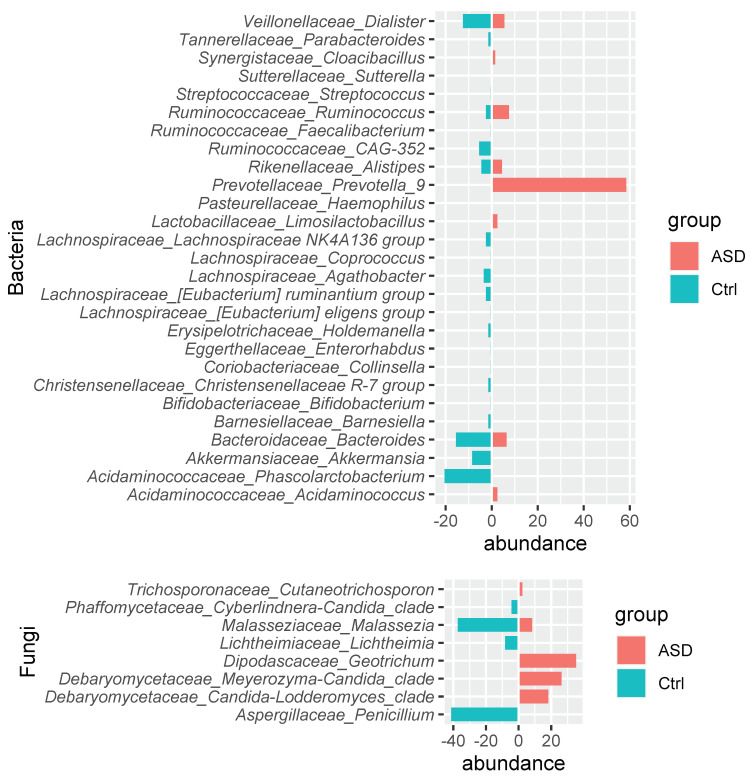
Bacteria (**up**) and fungi (**down**) genera relative abundance.

Due to high heterogeneity of the sample compositions (see Supplementary Figure S1), we performed the metataxonomic analysis comparing each ASD sample to the whole control set (*n* = 6) and thus evidenced that twenty bacteria families were prevalent in the most Ctrl samples but were not so frequent in ASD (Figure 3, Supplementary Supplementary Figure S2 and Table S1). These included the previously identified *Akkermansiace*, *Barnesiellaceae, Eggertellaceae,* and *Tannerellaceae* characterising Ctrl samples, while *Sutterellaceae* for the ASD samples and *Acidaminococaceae* and *Ruminococcaceae* were detectable in both sample types. Instead, several families displayed a different trend: *Bacterioidaceae*, *Bifidobacteriaceae*, *Christensellaceae*, *Erysipelotrichaceae Lachnospiraceae*, and *Streptococcaceae* were shown as characteristic of Ctrls, while *Pasturellaceae* and *Prevotellaceae* were detectable only in ASD. Moreover, *Coriobacteriaceae* and *Veillonellaceae* were not previously identified as well as *Lactobacillaceae* and *Synergisticaceae*, which were respectively under- and over- represented in ASD samples. Among these families, *Prevotella_9* and *Ruminococcus* genera are mainly present in ASD samples, while *Phascolarctobacterium, Akkermansia*, Bacteroides, *CAG-352,* and Dialister genera were principally detectable in Ctrl samples.

#### 3.1.2. Fungi Profiling: Metataxonomic Analysis

Although the 18S analysis was performed on the Fungi kingdom, in order to reduce the huge amount of vegetable sequence detected in these samples, low abundances can be detected in all samples. Moreover, the mycobiome comparative analysis returned even less variability in ASD samples. Only five fungi families can be significantly identified: *Saccharomycetaceae* and *Debariomycetaceae* in both sample types, while *Aspergillaceae*, *Malassenziaceae,* and *Cladosporaceae* are detectable only in controls (Figure 2b). Although *Saccharomycetaceae* was the most diffused family, it was not further classifiable at genus level. Among ASD, the *Debaryomycetace* family was predominant and represented by *Candida-Lodderomyces_clade* and *Meyerozyma-Candida_clade*. In neurotypical individuals, the predominant genera were *Penicillium* and *Malassezia*, belonging to *Aspergillaceae* and *Malasseziaceae*, respectively. 

The analysis performed comparing the mycobiome profile of each ASD sample to those of the whole Ctrl group (Supplementary Table S2) returned *Aspergillaceae* and *Malasseziaceae* as prevalent in control samples, while *Debaryomycetace* and *Dipodascaceae* were predominant in ASD samples (Figure 3). This analysis confirms for the *Debaryomycetace* family *Candida-Lodderomyces_clade* and *Meyerozyma-Candida_clade* genera and annexes *Geotrichum* from *Dipodascaceae* characterising ASD mycobiota and *Penicillium*, belonging to the *Aspergillaceae* family, detectable only in Ctrl samples, while *Malassezia* were seriously reduced in ASD samples.

### 3.2. sncRNA Profiling

The analysis of human sncRNAs in stool samples was performed by the bioinformatics pipeline as described in [49]. Quality control trimming and filtering returned sample reads in the average of 26.2 million and 15–51 base length. About 2.4 million reads per sample (9.2%) mapped to the human genome (GRCh38), and an average of 1.2 million reads per sample were assigned to ncRNA classes. Among these reads, 58.2% represented long ncRNAs, and 41.8% sncRNAs. The 0.5% fraction of ncRNA reads were miRNAs, according to previously published literature [61], and 9.6% piRNAs.

**Figure 4:**
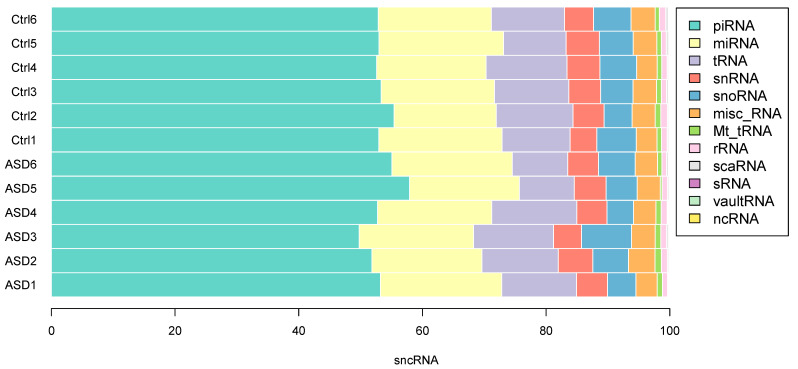
Distribution of the sncRNA fraction in stool samples. SncRNAs are equally distributed among samples from ASD patients (ASD1–6) and controls (CSV7–13). Data are expressed as percentage of the number of sncRNA per class.

We identified a total of 11,596 sncRNAs in stool samples, and no relevant differences were observed between cases and controls; a mean of 1271 piRNAs (53.4%) and 444 miRNAs (18.6%) per sample were detected. The distribution of the different classes of sncRNA did not differ between samples of ASD patients and controls (Figure 4). The most expressed miRNAs in both patients and controls are hsa-miR-182-5p and hsa-miR-681; hsa-miR-657 and hsa-miR-2110 are mainly present in all ASD, while hsa-miR-1203 is mainly present in neurotypical individuals. The most represented piRNAs in all samples are hsa-piR-27489, hsa-piR-32912, hsa-piR-32921, hsa-piR-23722, and hsa-piR-19705; in addition, piRNAs hsa-piR-28059 is highly expressed in ASD subjects, while hsa-piR-33182 and hsa-piR-33031 are highly expressed in healthy subjects.

As for the metataxonomic analysis, the sncRNA analysis was also performed comparing each ASD sample to the whole controls collection (*n* = 6) (Supplementary Table S3). A total of 42 miRNAs were up-regulated in three out of six ASD samples although none resulted common to all the three samples. Target gene analysis of the dysregulated miRNAs identified 18 target genes common to three ASD stool samples (Table 1). The differential expression analysis conducted on piRNAs returned no down-regulated piRNAs and a total of 84 up-regulated piRNAs in four out of six patients with ASD. Among these, only hsa-piR-21363 was common to two patients, while three target genes (CFLAR, GOLGA6L2, and SLC2A4) were common to four patients. Moreover, considering both miRNA and piRNA gene targets, five genes that resulted common in more than two samples (N4BP1, SLC2A4, SLC12A6, TTN, and ZNF33A) were identified. Table 1 summarises the 26 target genes of identified transcriptional modulators, considering the tissue expression, protein annotation, related disease, and SFARI score for proteins mutated in ASD. 

**Figure 5:**
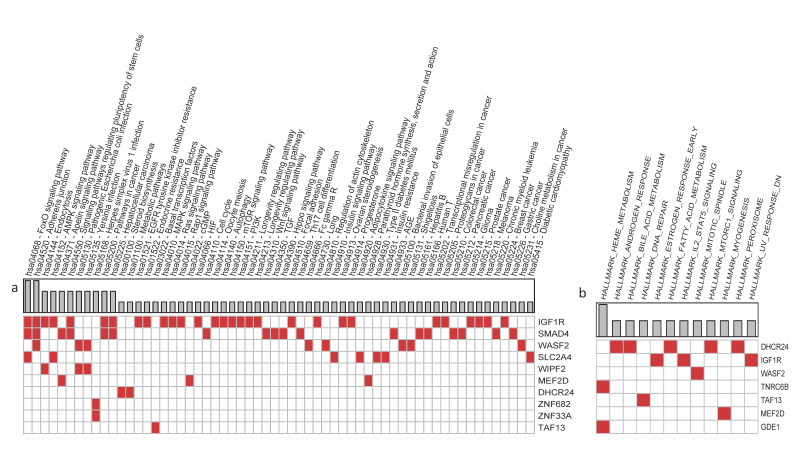
Functional annotation of miRNA and piRNA target genes from KEGG (**a**) and MSigDB-Hallmark (**b**).

The miRNA and piRNA target genes common to at least three samples were annotated with KEGG database and MSigDB-Hallmark gene sets. Results are displayed in Figure 5, grouped by pathway (a) or Hallmark gene set (b). This analysis returns pathways or biological processes involving one or more target genes. These are related to cell–cell junction, bacterial invasion, inflammation, and metabolite signalling and are pathways linked to autism.

### 3.3. Case Study: Analysis of Siblings

Within the analysed stool samples, four were obtained from two couples of siblings: couple #1 (male–male), one subject with ASD and the other neurotypical control, and couple #2, a male with ASD and a neurotypical female. We separately analysed the data from these samples to highlight differences of gut microbial community and sncRNAs between patient and control with common genetic background and similar diet.

**Figure 6:**
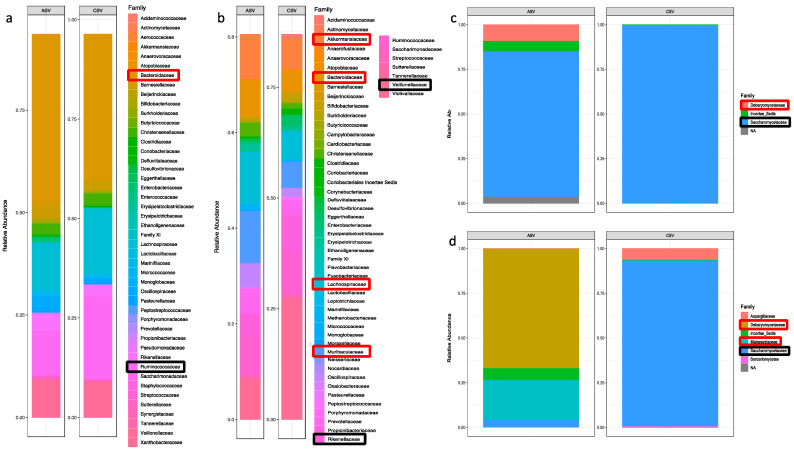
Microorganism composition of the couples of siblings. Microbiota composition, expressed as abundance fractions, in couple #1 (**a**) and in couple #2 (**b**); Mycobiota in (**c**) for couple #1 and in (**d**) couple #2. Families are red squared if increased in ASD sample and black squared if decreased.

The microbiota composition of the two couples of siblings was compared; abundance fractions are reported in Figure 6. Couple #1 displays a similar microbiota composition, and the only relevant differences concern the *Bacteroidaceae* and the *Rumicococcaceae* family (Figure 6a), which, respectively, increased and reduced in the ASD individual, in contrast to *Debaryomycetaceae* (Figure 6c), which was noticeable only in the ASD sibling mycobiota to the detriment of *Saccharomycetaceae*. A different composition was identified in the couple #2, with the ASD sibling displaying increased *Akkermansiaceae*, *Bacetroidaceae*, *Lachnospiraceae,* and *Muriobaculaceae*, while *Rikenellaceae* and *Veillonellaceae* were decreased in the ASD individual (Figure 6b). The mycobiota composition displays a relevant increase in *Debaryomycetaceae* and *Malasseziaceae* in ASD to the detriment of *Saccharomycetaceae* (Figure 6d). 

By comparing the results obtained within each couple of siblings, we identified up-regulated and down-regulated miRNAs and piRNAs by statistical analyses and defined as statistically significant those ncRNA with log2 fold change ≤ −1 or ≥ 1 and *p*-value < 0.05 (Fisher’s test) (Supplementary Table S4). A total of 750 miRNAs were identified in couple #1, and among these, 93 have a significantly differential expression. Among these, 60 miRNAs were detected in both individuals of couple #1, 32 were up-regulated, and 28 down-regulated in ASD sample. Moreover, 15 miRNAs were detected only in the ASD subject and 18 only in the healthy sibling. The top three down-regulated miRNAs in ASD sample are hsa-mir-937, hsa-mir-3197, and hsa-mir-103a-1, while those up-regulated are hsa-mir-4700, hsa-miR-657, and hsa-mir-2110. A total of 2177 piRNAs were identified in stool samples from couple #1, and 329 were significantly dysregulated. Among these, 112 are up-regulated and 52 down-regulated and present in both subjects belonging to couple #1, whereas 109 are present only in the stool sample from the ASD subject and 56 in sample from the healthy sibling (for details, see Table 2). The top three down-regulated piRNAs in ASD are hsa-piR-6691, hsa-piR-6693, and hsa-piR-29205, while hsa-piR-28269, hsa-piR-32987, and hsa-piR-28059 are significantly up-regulated.

In couple #2, we identified 581 miRNAs and 1912 piRNAs. There are 51 significantly differentially expressed miRNAs common to both samples, 11 up-regulated and 40 down-regulated in the ASD sample. Moreover, 19 miRNAs were identified only in the ASD sample and 23 only in the control sibling. In couple #2, 222 piRNAs significantly differentially expressed and common to both samples were identified. Among these, 66 were up- and 256 down-regulated in ASD sample, 33 detected only in ASD, and 85 only in sample from the neurotypical sister.

The comparison between couple #1 and #2 reveals that there are three common miRNAs down-regulated in samples from ASD, namely hsa-miR-10b-5p, hsa-miR-22-3p, and hsa-miR-192-5p, and four up-regulated, namely hsa-miR-6760-5p, hsa-mir-6766, hsa-mir-6839, and hsa-mir-3976. The common dysregulated piRNAs are 44, down-regulated 13, and up-regulated 31 (for details see Table 3). 

## 4. Discussion

The role of gut microbial composition and interactions among the microbial strains as well as the bidirectional communication between the host and gut microbiota are continuously emerging in health and disease [13]. Faeces contain much information useful to shed the light on gut microorganisms and the molecular trafficking involved in the host–microbiota cross-talk. For these reasons, faeces samples should be analysed as a whole, at the microbial and molecular level. Nevertheless, most studies about gut microbiota only refer to the prokaryotic component, and the role of eukaryotes and host–microbiota communication is poorly investigated. Thanks to the progress achieved in present years regarding the high-throughput sequencing technologies and in the computational analysis, due to the data generated by these technologies, we produced the eukaryotic and prokaryotic profile as well as the host transcriptional modulator profile (miRNAs and piRNAs) gathered in the faeces collected from a small group of children with ASD or neurotypical development. 

Considering bacterial composition, we found a dysbiosis consisting of lower alpha diversity in ASD stools. This is in line with the literature that associates this index with the severity of social impairment, while it is independent of IQ [10,18]. We found that *Firmicutes* and *Bacteroidetes* were the most abundant phyla in both ASD and Ctrl faeces samples, and the *Bacteroidetes/Firmicutes* ratio was higher in ASD samples than in Ctrl, mainly due to decrease of *Firmicutes.* The imbalance in this ratio is still known in literature [72]; however, some evidence sustains that the *Bacteroidetes/Firmicutes* ratio was higher in ASD samples than in Ctrl [59,60], while other studies claim otherwise [33,73]. Due to this inconsistency, the role of *Bacteroidetes/Firmicutes* remains controversial.

At the genera taxonomic level, the *Acidaminococcus* enrichment and *Dialister* depletion that we observed in ASD is in line with previous articles [33,74]. Instead, *Prevotella_9* and the overall increase in the *Prevotella* family is arguably reported in ASD children [74,75]. Moreover, an increase in *Ruminococcus* and *Streptococcus* and a decrease in *Agathobacter* were previously associated to ASD [59,76] and negatively associated with sleep and language disorders [77]. In line with our results, the presence of *Alistipes* in ASD, or more generally in patients with neurodevelopmental disorders, has also been discussed [31,33,78]; *Alistipes* probably has a role in decreasing serotonin availability, destabilising the gut–brain axis. Finally, literature reports *Christensenellaceae* as signature of a healthy gut [79,80]. Furthermore, *Akkermansia* is related to a healthy gut, and it is thought to have anti-obesity and anti-diabetic effects [81,82]; this family promotes gut barrier integrity, modulates immune response, and inhibits inflammation and syntrophy with other microbiota species [83]. *Akkermansia* is evidenced as mucolytic bacteria, and its lower abundance (or absence) in ASD supports the hypothesis of possible mucus barrier in children with autism [60,84]. Besides, short chain fatty acid (SCFA) produced by several bacteria families are involved in the release of mucus, with the exception for succinate not consumed by *Phascolarctobacterium* [85] in ASD cohort children. Bacteria families involved in SCFA production were prevalently identified in controls, namely *Akkermansia* [86] *Phascolarctobacterium*, *Lachnospiraceae,* and *Agathobacter*, a producer of butyrate and beneficial SCFA [77,87,88,89]. However, recent evidence suggests an involvement of acetate and propionate in various disorders, including obesity [90]. Overall, SCFAs play a modulatory role in the microbiota–gut–brain axis, mediating behaviour and intestinal physiology [91]. In particular, butyrate, the levels of which are known to decreased in ASD [92], has multiple effects on the gut–brain axis, anti-inflammatory, blood-brain barrier, and gut-permeability regulators [93] and on healthy gut physiology, preventing pathogen invasion, modulating the immune system, and reducing cancer progression [94].

Furthermore, for the fungi kingdom, the mycobiome comparative analysis identified only *Saccharomycetaceae* and *Debariomycetaceae* families in both ASD and Ctrl groups, while *Aspergillaceae* (including *Penicillium)* and *Malassenziaceae* were found only in neurotypical according to literature [33,95]. The *Malassezia* role in the healthy gut is still unclear: it is correlated with neurological disease [96], but it represents a large amount of breast milk mycobiota, and it is found in healthy faecal samples [97]. In ASD, instead, only the *Debaryomycetace* family was predominant and consists of *Candida-Lodderomyces_clade* and *Meyerozyma-Candida_clade* that have never been reported before in ASD or in any other gut microbiota study. Although fungi are poorly studied, an important presence of *Candida* among ASD gut prokaryotes was reported, and anti-*Candida albicans* IgG antibodies were also detected in the plasma of children with ASD, making genus *Candida* a new microbial risk factor for the condition [33,98]. *Candida,* when overgrown, produces root-like structures that penetrate the intestinal wall, causing the leaky-gut syndrome. This allows macromolecules, such as toxins and food antigens, to enter the bloodstream and trigger food intolerance and allergies, as described in a subset of children with ASD [99].

We investigated sncRNA by next-generation sequencing technologies, with the advantage of looking for all sncRNA classes. Indeed, previous studies about sncRNAs in ASD were performed by quantitative real-time PCR or arrays technologies that investigate only a relatively small panel of target miRNAs. Therefore, these studies may return a focused profile, while a complete and unbiased sncRNA view, such as that obtained by sncRNA-seq analysis, is needed. Indeed, as emerged by our results, some sncRNAs that we found over-expressed in ASD target genes and pathways involved in inflammation and intestinal permeability, while others are involved in ASD. Due to the nature of investigated samples, we are unable to define if these latter sncRNAs are limited to stool and act only at gastrointestinal level or pass into the bloodstream and act at systemic level, too. The most represented miRNAs in all ASD are hsa-miR-2110 and hsa-miR-657. The first one was found in EV released from mesenchymal stem cells [100], up-regulated in serum exosome from patients with glioblastoma [101], supporting the hypothesis that hsa-miR-2110 is released into the lumen. The increased expression of hsa-miR-657 is associated to inflammatory response in gestational diabetes mellitus [102]. Interestingly, maternal gestational diabetes mellitus increases the risk factor for autism in offspring [103]. 

Host miRNAs are able to enter bacteria and regulate their gene expression and growth [34]. However, our results cannot be compared to those of the literature because the study is limited to a small number of miRNAs on *Fusobacterium nucleatum* and *E. coli* [34].

In our study, piRNAs are the most represented sncRNA. This is of particular interest because, although the role of piRNAs is still emerging, they have never been investigated so far in the faeces of individuals with ASD. PiRNAs data refer mainly to germline or are related to cancer cells [104,105] or faeces of patients with colorectal cancer [61]. They were detected in EVs in different body fluids [106,107], so we speculate that the piRNAs we found in stools may have been released into the gut lumen and may interact also with micro- and mycobiota. 

Considering the miRNA and piRNA target genes common to multiple samples, four were already present in SFARI database: *NACC1*, *SMAD4*, *TNRC6B,* and *TTN,* and their mutants are implicated in ASD and other neurodevelopmental disorders (See Table 1). Besides, CNV in locus 9p24.3, overlapping *CBWD1,* was identified in autistic patients [108,109,110]. Numerous genes are also involved in mental retardation, schizophrenia, and other neurodevelopmental disorders (see Table 1 for details). We suppose that the up-regulated miRNAs and piRNAs can negatively influence the expression of these genes and therefore dysregulate the biological pathways involved in neurodevelopment. SLC2A4 is a glucose transporter whose impairment has again been associated with diabetes mellitus [111]. N4BP1 is a potent suppressor of cytokine production, and thus, its inhibition increases innate immune signalling and inflammation [112]. Finally, HSBP1 and IGF1R were found differentially expressed in autism [113,114,115,116,117] and, directly or indirectly, modulated by microbiota [118,119]; namely, HSBP1 is down-regulated after antibiotic administration, and IGF-1 level is affected by gut microbiota composition.

These 26 genes belong to pathways that are, directly or indirectly, associated with ASD [69] or its comorbidities. For instance, *IGF1R*, *SMAD4*, and *WASF2* genes are involved in “Adherens junction” KEGG pathway and therefore could induce an imbalance in intestinal tight junctions. Interestingly, this pathway could also be implicated in celiac immune response [120], a condition common in ASD. WASF2 is also involved in “bacterial invasion of epithelial cells” and in “regulation of actin cytoskeleton” pathways, controlling cellular actin dynamics [121]. Then again, “Adipocytokine signalling pathway” is involved in cytokine production and inflammation as well as “TGF- β signalling”. This latter is emerging as a key regulator of nervous system physiology also involved in nervous system diseases and injury [122]. Moreover, pathways directly connected with ASD are: “Long term depression” that, together with “Long-term potentiation”, are the key regulators of long-lasting synaptic plasticity at the basis of learning and memory and whose impairment is involved in many brain disorders, including ASD [123,124]; “Insulin signalling pathway” promotes neuronal circuit development and maturation to an extent that, since its safety/preliminary efficacy for the treatment of Rett syndrome has emerged, clinical trials to ameliorate ASD symptoms are ongoing [125].

Similarly, Hallmark analysis identified “Apical surface”, “Fatty acid metabolism”, and “IL2 STAT5 signaling” hallmarks. The first one refers to a group of proteins expressed on the apical surface of epithelial cells, including enterocytes, that play a crucial role in cell polarity and thus act together with tight junction proteins in maintenance of epithelial tissue integrity [126]. Instead, the “Fatty acid metabolism” gene set includes genes known to be affected by gut microbiota composition [127]: saturated fatty acids increase bile-tolerant bacteria and reduce microbial diversity, while unsaturated fatty acids, such as omega-3, exert anti-inflammatory activity that contributes to gut health. Thus, an overall diet scheduling fat intake to alleviate gastro-intestinal disorders has been suggested [128]. Finally, IL2/STAT5 signalling pathway has a role in differentiation and homeostasis of both pro- and anti-inflammatory T cells, determining the molecular details of immune regulation [129,130].

The limit of this study is the small size of samples; nevertheless, this is the first study that integrates metagenomic data with small ncRNA profile to investigate the host–gut microbiota cross-talk in ASD and that detects and analyses the piRNA profile in stools from individuals with ASD.

## 5. Conclusions

Since multiple interactions occur between host and gut microorganisms as well as among different microbial strains, faecal samples represent a source of information that should be studied as a whole. A complex picture emerges from this study due to the abundance and variability of gut microbiota and to the heterogenicity of samples. Based on our results and those of literature, we tried to find a relationship between the dysregulated gut microbial strains and up-regulated sncRNAs in ASD. We identified novel fungi genera and confirmed the bacteria dysbiosis, highlighting the possible role of dysregulated microbiome metabolites in ASD aetiology, too. Moreover, for the first time, we profiled miRNAs and piRNAs in ASD stool samples, which targeted pathways with roles in ASD. Disentangling the host–microbiome cross-talk is crucial to understand the role of dysbiosis in ASD onset and to design diagnostic tools and personalised therapeutic interventions. Host–gut microbiota cross-talk in health and disease, mediated by small ncRNAs, needs further insights, and omic and multi-omic data integration studies play a key role for these purposes [131,132].

The results obtained should be confirmed in large ASD and Ctrl cohorts, and the effect of the up-regulated miRNAs and piRNAs should be studied on microbial cultures as well. 

## Figures and Tables

**Table 1:** List of miRNAs, piRNAs and miRNA + piRNAs common targets. “Single-cell type specificity” was obtained by single-cell transcriptomics; “Tissue specificity (RNA)” and “GI RNA expression” were obtained by RNA-seq; “GI protein expression” was obtained by immunocytochemistry investigations. All these data are from ProteinAtlas (https://www.proteinatlas.org, accessed on 2 December 2021). Biological process and Molecular function data are from UniProt (https://www.uniprot.org, accessed on 2 December 2021). SFARI score categories are reported in the following website (https://gene.sfari.org/about-gene-scoring/, accessed on 2 December 2021). Abbreviation: GI, gastrointestinal; ASD, autism spectrum disorders.

Small ncRNAs		Target Gene	Tissue Expression Cluster (RNA)	Single-Cell Type Specificity (Enhanced in)	Tissue Specificity (RNA)	GI RNA Expression (Score)	GI Protein Expression (Score)	Biological Process	Molecular Function	Autism-Related Disorders	SFARI (Score Categories)
**miRNA**	hsa-miR-182-3p hsa-miR-99a-5p hsa-miR-4758-5p	*CBWD1*	Intestine—Vesicular transport	Nonspecific	Low	Low	Medium-low	NA	ATP binding		
	hsa-miR-3911 hsa-miR-99a-5p hsa-miR-595	*DHCR24*	Non-specific—Unknown function	Hepatocytes, Alveolar cells type 2, Theca cells, Alveolar cells type 1	Adrenal gland, liver	Low	NA	Cholesterol biosynthesis, Cholesterol metabolism, Lipid biosynthesis, Lipid metabolism, Steroid biosynthesis, Steroid metabolism, Sterol biosynthesis, Sterol metabolism	Oxidoreductase	Desmosterolosis (OMIM 602398) [62]	
	hsa-miR-4674 hsa-miR-4494 hsa-miR-6841-3p hsa-miR-99a-5p hsa-miR-4487 hsa-miR-3613-3p	*GDE1*	Non-specific—Mitochondria	Syncytiotrophoblasts	Low	High	NA	Lipid metabolism	Hydrolase		
	hsa-miR-4742-3p hsa-miR-5689 hsa-miR-766-3p	*HSBP1*	Non-specific—Mitochondria	Respiratory epithelial cells	Low	High	NA	Negative regulator of the heat shock response	Identical protein binding; transcription corepressor activity		
	hsa-miR-182-5p hsa-miR-96-5p hsa-miR-99a-5p hsa-miR-3613-3p hsa-miR-8071	*IGF1R*	Ciliated cells—Cilium assembly	Oligodendrocytes, microglial cells, excitatory neurons, oligodendrocyte precursor cells, inhibitory neurons	Low	Medium	High	Host-virus interaction	Kinase, receptor, transferase, tyrosine-protein kinase		
	hsa-miR-4712-3p hsa-miR-1324 hsa-miR-99a-5p	*MEF2D*	Non-specific—Translation	Cone photoreceptor cells, sertoli cell; cluster in intestinal epithelial cells	Skeletal muscle	Low	High	Apoptosis, differentiation, neurogenesis, transcription, transcription regulation	Activator, developmental protein, DNA-binding		
	hsa-miR-4712-3p hsa-miR-6865-5p hsa-miR-3911 hsa-miR-595 hsa-miR-2110 hsa-miR-144-3p hsa-miR-3615	*NACC1*	Non-specific—Unknown function	Non-specific	Low	Medium	Medium	Transcription, transcription regulation	Repressor	Disease mutation, epilepsy, mental retardation [63]	1S
	hsa-miR-4712-3p hsa-miR-5689 hsa-miR-96-3p	*OLA1*	Non-specific—Mitochondria	Non-specific; cluster in smooth muscle cells	Low	Medium	High	ATP metabolic processes	Hydrolase		
	hsa-miR-4712-3p hsa-miR-4742-5p hsa-miR-99a-5p hsa-miR-144-3p hsa-miR-182-5p hsa-miR-96-5p hsa-miR-766-3p	*RPL7L1*	Non-specific—Mitochondria	Non-specific	Low	Medium	NA	Blastocyst formation; maturation of LSU-rRNA from tricistronic rRNA transcript (SSU-rRNA, 5.8S rRNA, LSU-rRNA)	Ribonucleoprotein, ribosomal protein		
	hsa-miR-182-5p hsa-miR-144-3p hsa-miR-933 hsa-miR-154-5p	*SMAD4*	Non-specific—Translation	Granulosa cells	Low	Medium	High	Transcription, transcription regulation	DNA-binding	Myhre Syndrome [64]: Juvenile polyposis syndrome	2
	hsa-miR-4487 hsa-miR-766-3p hsa-miR-144-3p hsa-miR-99a-5p	*SMARCA5*	Immune cells—Transcription, translation	Alveolar cells type 1	Low	Medium	High	Host-virus interaction	Chromatin regulator, helicase, hydrolase	Neurodevelopmental syndrome [65]	
	hsa-miR-3613-3p hsa-miR-766-3p hsa-miR-5689 hsa-miR-96-3p hsa-miR-144-3p	*TAF13*	Non-specific—Translation	Suprabasal keratinocytes; cluster in macrophages	Low	Medium	Medium	Transcription, transcription regulation	DNA binding	Mental retardation, autosomal recessive 60 (OMIM 617432), Autosomal-Recessive Intellectual Disability [66]	
	hsa-miR-4742-5p hsa-miR-99a-5p hsa-miR-2113 hsa-miR-766-5p	*TNRC6B*	Bone marrow, brain—smell perception, nucleosome	Non-specific	Low	Low	High	RNA-mediated gene silencing, translation regulation	RNA-binding	Complex neurodevelopmental disorder involving spoken language, intellectual disability, neurobehavioural phenotype (ASD), and epilepsy [67,68,69]	2
	hsa-miR-4742-3p hsa-miR-1324 hsa-miR-5689 hsa-miR-6865-3p	*UHMK1*	Non-specific—Unknown function	Non-specific	Low	Medium-high	NA	Neuron projection development	Kinase, RNA-binding, serine/threonine-protein kinase, transferase	Schizophrenia [70,71]	
	hsa-miR-3613-3p hsa-miR-766-3p hsa-miR-3615	*WDR12*	Non-specific—Mitochondria	Non-specific; cluster in Smooth muscle cells	Low	Medium-high	Medium-high	Ribosome biogenesis, rRNA processing	Ribonucleoprotein complex binding		
	hsa-miR-3613-3p hsa-miR-99a-3p hsa-miR-6939-5p hsa-miR-4758-3p hsa-miR-766-5p	*WIPF2*	Bone marrow—Differentiation	Non-specific; cluster in intestinal epithelial cells	Low	High	High	Actin filament-based movement	Actin-binding		
	hsa-miR-766-3p hsa-miR-3615 hsa-miR-4712-5p hsa-miR-595	*ZNF682*	Skin—Unknown function	Oligodendrocytes	Low	Medium-low	NA	Transcription, transcription regulation	DNA-binding		
	hsa-miR-96-3p hsa-miR-5689 hsa-miR-2110 hsa-miR-6760-5p	*ZNF703*	Striated muscle—Muscle contraction	Syncytiotrophoblasts	Skeletal muscle	Medium-low	Medium	Transcription, transcription regulation	Repressor		
**piRNA**	hsa-piR-16407 hsa-piR-18524	*CFLAR*	Lung—Lung homeostasis	Langerhans cells, urothelial cells; cluster in macrophages	Low	Low	High	Apoptosis, host-virus interaction	Cysteine-type endopeptidase activity involved in apoptotic signalling pathway		
	hsa-piR-21363	*GOLGA6L2*	Testis—Meiosis	Early spermatids	Testis	NA	NA	NA	NA		
	hsa-piR-21363	*SLC2A4*	Striated muscle—Muscle contraction	Cardiomyocytes	Heart muscle, skeletal muscle	Low	NA	Transcription, transcription regulation	DNA-binding		
**mirRNA**/ **piRNA**	hsa-miR-708-3p hsa-miR-766-3p hsa-piR-9505	*N4BP1*	Skin—Epithelial junctions	Alveolar cells type 1, glandular and luminal cells, cluster in endometrium	Low	Medium	High	Immunity, innate immunity	Hydrolase, nuclease, RNA-binding		
	hsa-miR-766-5p hsa-piR-21363	*SLC2A4*	Striated muscle—Muscle contraction	Cardiomyocytes	Heart muscle, skeletal muscle	Low	NA	Transcription, transcription regulation	DNA-binding		
	hsa-miR-5689 hsa-piR-2001	*SLC12A6*	Immune cells—Transcription, Translation	Cone photoreceptor cells, rod photoreceptor cells; cluster in B-cells	Low	Low	Medium	Ion transport, potassium transport, symport, transport	potassium:chloride symporter activity	Andermann syndrome (OMIM #218000)	
	hsa-miR-144-3p hsa-piR-13910	*TTN*	Striated muscle—Muscle contraction	Cardiomyocytes	Skeletal muscle, tongue	Very low	NA	Cardiac muscle tissue morphogenesis, skeletal muscle thin filament assembly	Calmodulin-binding, Kinase, serine/threonine-protein kinase, transferase		3S
	hsa-miR-3911 hsa-miR-4487 hsa-piR-433	*ZNF33A*	Non-specific—Transport via ER	Non-specific	Low	Medium	Low	Transcription, transcription regulation	DNA-binding		

**Table 2:** MiRNAs and piRNAs count in the couples of siblings. Within the parentheses are significantly different data (Fisher tests, *p* < 0.05); Ctrl, control.

		Common to ASD and Ctrl			Only in	
		Total	Significantly Up-Regulated	Significantly Down-Regulated	ASD	Ctrl
miRNA	Couple #1	207 (60)	32	28	226 (15)	317 (18)
	Couple #2	152 (51)	11	40	245 (19)	184 (23)
piRNA	Couple #1	380 (164)	112	52	787 (109)	1010 (56)
	Couple #2	509 (222)	66	156	757 (33)	617 (85)

**Table 3:** MiRNAs and piRNAs common to the couples #1 and #2. log2FC, log2 fold change; ASD, subject with ASD; Ctrl, neurotypical subject; Fisher_p, Fisher’s test *p*-value.

	Couple #1				Couple #2			
Gene Name	ASD	Ctrl	log2FC	Fisher_p	ASD	Ctrl	log2FC	Fisher_p
hsa-miR-10b-5p	27.1	79.7	−1.52	2.9 × 10^−7^	0.0	29.7	−4.94	2.0 × 10^−9^
hsa-mir-192	33.2	94.1	−1.48	5.7 × 10^−8^	0.3	8.0	−2.77	7.8 × 10^−3^
hsa-miR-22-3p	11.3	28.7	−1.26	6.4 × 10^−3^	0.0	7.2	−3.04	1.6 × 10^−2^
hsa-miR-192-5p	90.8	185.4	−1.02	1.6 × 10^−8^	0.0	20.8	−4.45	9.5 × 10^−7^
hsa-miR-6760-5p	7.9	0.9	2.22	3.9 × 10^−2^	11.4	0.0	3.63	9.8 × 10^−4^
hsa-miR-6766	23.6	1.8	3.14	1.0 × 10^−5^	27.0	0.0	4.81	1.5 × 10^−8^
hsa-miR-6839	7.9	0.0	3.15	7.8 × 10^−3^	113.3	0.0	6.84	0.0 × 10^0^
hsa-miR-3976	14.8	0.0	3.99	6.1 × 10^−5^	36.1	14.4	1.27	2.6 × 10^−3^
hsa-piR-28021	0.0	429.1	−8.75	0.0 × 10^0^	0.3	57.7	−5.47	0.0 × 10^0^
hsa-piR-8876	0.0	46.6	−5.57	0.0 × 10^0^	0.0	9.6	−3.41	2.0 × 10^−3^
hsa-piR-12132	0.0	43.9	−5.49	0.0 × 10^0^	14.3	804.0	−5.72	0.0 × 10^0^
hsa-piR-32989	0.0	9.9	−3.44	2.0 × 10^−3^	2.3	37.7	−3.56	1.0 × 10^−9^
hsa-piR-5819	0.0	7.2	−3.03	1.6 × 10^−2^	0.7	8.8	−2.57	2.1 × 10^−2^
hsa-piR-14261	612.0	3592.7	−2.55	0.0 × 10^0^	9.1	20.8	−1.11	4.3 × 10^−2^
hsa-piR-33186	4681.1	23,105.1	−2.30	0.0 × 10^0^	0.0	5260.6	−12.36	0.0 × 10^0^
hsa-piR-33033	6091.9	20,508.4	−1.75	0.0 × 10^0^	2.9	1094.1	−8.12	0.0 × 10^0^
hsa-piR-5751	291.6	907.4	−1.63	0.0 × 10^0^	0.0	32.1	−5.05	0.0 × 10^0^
hsa-piR-8213	11.3	28.7	−1.26	6.4 × 10^−3^	1.0	8.0	−2.19	3.9 × 10^−2^
hsa-piR-32837	413.8	944.1	−1.19	0.0 × 10^0^	1.6	74.5	−4.85	0.0 × 10^0^
hsa-piR-32914	413.8	944.1	−1.19	0.0 × 10^0^	1.6	74.5	−4.85	0.0 × 10^0^
hsa-piR-28066	1331.4	2873.5	−1.11	0.0 × 10^0^	1.0	40.9	−4.41	0.0 × 10^0^
hsa-piR-31090	39.3	18.8	1.02	1.2 × 10^−2^	38.4	16.8	1.14	6.4 × 10^−3^
hsa-piR-32953	534.3	253.5	1.07	0.0 × 10^0^	588.9	8.0	6.03	0.0 × 10^0^
hsa-piR-26659	267.1	121.8	1.13	0.0 × 10^0^	608.1	283.0	1.10	0.0 × 10^0^
hsa-piR-21363	433.9	137.0	1.66	0.0 × 10^0^	2058.3	484.9	2.08	0.0 × 10^0^
hsa-piR-31508	18.3	4.5	1.82	4.3 × 10^−3^	35.2	2.4	3.41	1.0 × 10^−8^
hsa-piR-16407	14.0	1.8	2.42	4.2 × 10^−3^	230.8	10.4	4.34	0.0 × 10^0^
hsa-piR-2750	280.2	45.7	2.59	0.0 × 10^0^	121.4	16.8	2.78	0.0 × 10^0^
hsa-piR-30491	68.1	8.1	2.93	0.0 × 10^0^	157.9	55.3	1.50	0.0 × 10^0^
hsa-piR-22093	7.0	0.0	3.00	1.6 × 10^−2^	24.7	3.2	2.61	2.7 × 10^−5^
hsa-piR-18568	7.0	0.0	3.00	1.6 × 10^−2^	13.0	0.8	2.96	1.8 × 10^−3^
hsa-piR-21890	7.9	0.0	3.15	7.8 × 10^−3^	27.0	4.0	2.48	3.4 × 10^−5^
hsa-piR-13475	18.3	0.9	3.35	7.6 × 10^−5^	39.4	12.0	1.63	2.0 × 10^−4^
hsa-piR-8932	66.3	5.4	3.40	0.0 × 10^0^	86.6	40.1	1.09	3.7 × 10^−5^
hsa-piR-26586	9.6	0.0	3.41	2.0 × 10^−3^	10.1	0.0	3.47	2.0 × 10^−3^
hsa-piR-12718	9.6	0.0	3.41	2.0 × 10^−3^	232.1	46.5	2.30	0.0 × 10^0^
hsa-piR-33000	9.6	0.0	3.41	2.0 × 10^−3^	560.5	68.1	3.02	0.0 × 10^0^
hsa-piR-30677	11.3	0.0	3.63	9.8 × 10^−4^	75.2	6.4	3.36	0.0 × 10^0^
hsa-piR-33037	14.8	0.0	3.99	6.1 × 10^−5^	16.6	4.0	1.81	7.2 × 10^−3^
hsa-piR-24148	18.3	0.0	4.27	7.6 × 10^−6^	44.3	0.0	5.50	0.0 × 10^0^
hsa-piR-3308	18.3	0.0	4.27	7.6 × 10^−6^	21.2	4.0	2.15	9.1 × 10^−4^
hsa-piR-2934	73.3	2.7	4.33	0.0 × 10^0^	197.9	56.1	1.80	0.0 × 10^0^
hsa-piR-33019	20.1	0.0	4.40	1.9 × 10^−6^	392.3	0.0	8.62	0.0 × 10^0^
hsa-piR-3864	60.2	1.8	4.46	0.0 × 10^0^	99.3	4.0	4.32	0.0 × 10^0^
hsa-piR-25822	22.7	0.0	4.57	2.4 × 10^−7^	108.7	28.1	1.92	0.0 × 10^0^
hsa-piR-11291	46.3	0.9	4.64	0.0 × 10^0^	123.0	45.7	1.41	3.0 × 10^−9^
hsa-piR-4194	48.9	0.9	4.72	0.0 × 10^0^	59.9	24.8	1.24	1.9 × 10^−4^
hsa-piR-9502	26.2	0.0	4.77	3.0 × 10^−8^	22.5	0.0	4.55	4.8 × 10^−7^
hsa-piR-8096	27.9	0.0	4.86	7.0 × 10^−9^	83.0	11.2	2.78	0.0 × 10^0^
hsa-piR-30323	634.7	19.7	4.94	0.0 × 10^0^	595.1	8.0	6.05	0.0 × 10^0^
hsa-piR-32334	34.0	0.0	5.13	0.0 × 10^0^	15.6	0.0	4.06	3.1 × 10^−5^
hsa-piR-892	151.9	0.0	7.26	0.0 × 10^0^	18.6	3.2	2.22	8.5 × 10^−4^

## Data Availability

The data presented in this study are openly available in SRA (accession PRJNA813424) at the following link https://www.ncbi.nlm.nih.gov/sra/PRJNA813424, (accessed on 26 November 2021) and in GEO (accession GSE198199) at https://www.ncbi.nlm.nih.gov/geo/query/acc.cgi?acc=GSE198199, (accessed on 26 November 2021).

## Supplementary Materials

The following supporting information can be downloaded at: https://www.mdpi.com/article/10.3390/nu14071340/s1, Supplementary Figure S1: Relative abundances of 16S families; Supplementary Figure S2: Den-drograms of identified bacteria in ASD and Ctrl samples; Supplementary Table S1: 16S identified in faeces samples; Supplementary Table S2: 18S identified in faeces samples; Supplementary Table S3: sncRNAs identified in faeces samples; Supplementary Table S4: sncRNAs identified in the two couples of siblings.
