# Supplementary material for: Analysis of Faecal Microbiota and Small ncRNAs in Autism: Detection of miRNAs and piRNAs with Possible Implications in Host–Gut Microbiota Cross-Talk
Source: Nutrients. 2022 Mar 23;14(7):1340. doi: 10.3390/nu14071340 (PMC9000903; doi:10.3390/nu14071340)
Supplement: Supplementary file 1 [file nutrients-14-01340-s001.zip › Chiappori_Cupaioli_Supplementary Figures_accepted.pdf]

Analysis of faecal microbiome and small ncRNAs in autism: detection of miRNAs and piRNAs with possible implications in host-gut microbiota cross talk

Federica Chiappori†, Francesca Anna Cupaioli†, Arianna Consiglio, Noemi Di Nanni, Ettore Mosca, Vito Flavio Licciulli, and Alessandra Mezzelani*

Supplementary Figure

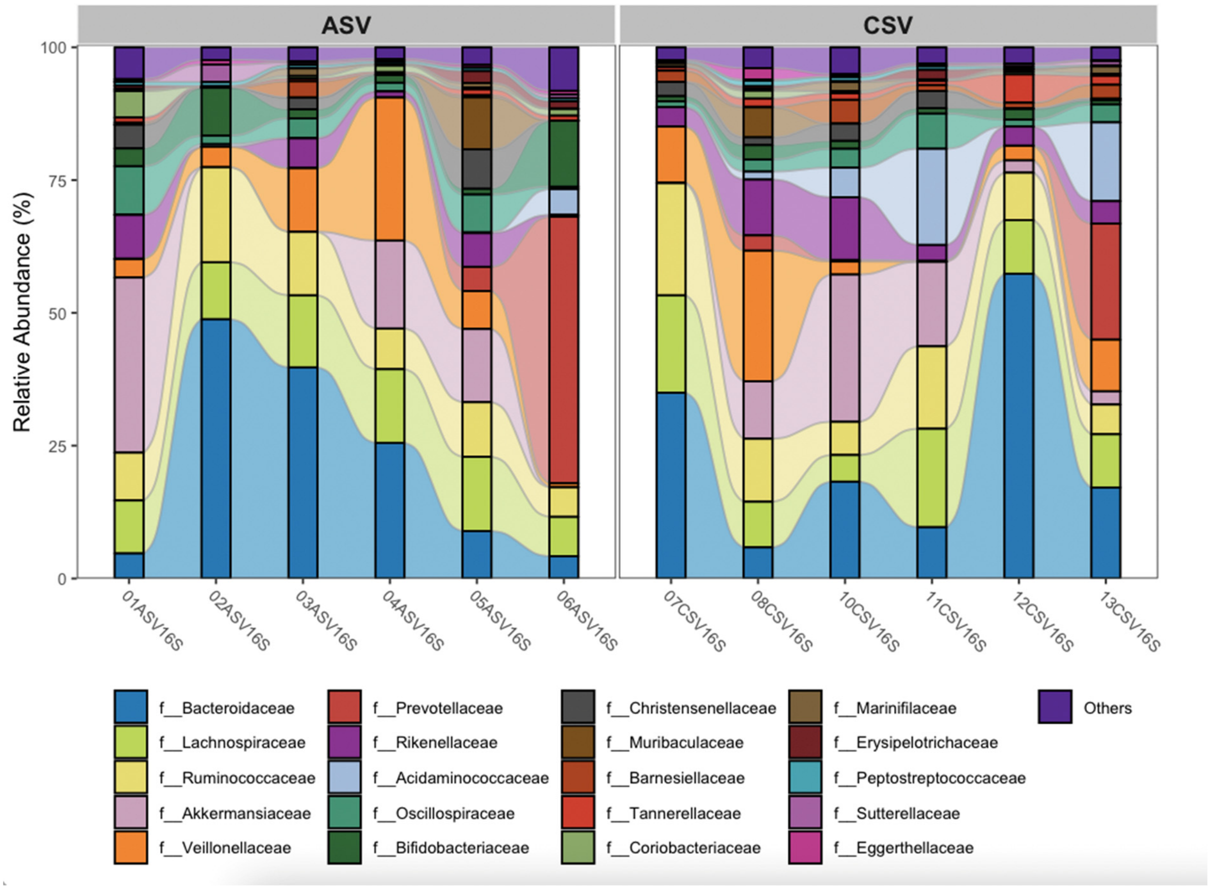

Supplementary Figure S1. Relative abundances of 16S families. This figure represents the high variability among the samples. ASV is referred to ASD; CSV is referred to Ctrl.

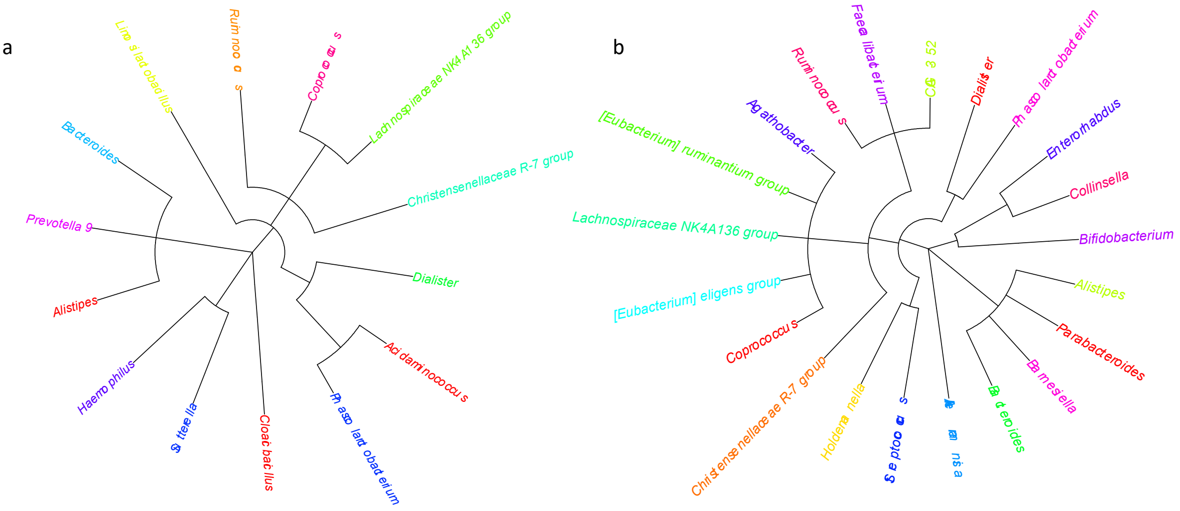

Supplementary Figure S2. Dendrograms of identified bacteria in ASD (a) and Ctrl (b) samples.

Supplementary tables caption

Supplementary table S1. 16S identified in faeces samples. Differential analysis was performed comparing ASD samples to the entire controls set (n=6).

Supplementary table S2. 18S identified in faeces samples. Differential analysis was performed comparing ASD samples to the entire controls set (n=6).

Supplementary table S3. sncRNAs identified in faeces samples. Differential analysis was performed comparing ASD samples to the entire controls set (n=6). sncRNAs with FDR < 0.05 were reported.

Supplementary table S4. sncRNAs identified in the two couples of siblings. Differential analysis was performed for each couple. sncRNAs with FDR < 0.05 were reported.
